# Medical Practice and the Companies Act

**Published:** 1907-04-13

**Authors:** 


### Medical Practice and the Companies Act.

It seems at first sight the statement of an absurd proposition, to say that an act which is illegal for any one individual, may be perfectly within the compass of the law if only it is performed by seven individuals acting in association. Yet such, it would appear, is the exact position in reference to the adoption of titles commonly employed to indicate the possession of a registrable medical or surgical qualification. Such titles, the legislature has decreed, may not be assumed except by those who have satisfied certain conditions in reference to education, examination, and registration. Any person who, not having satisfied these conditions, falsely assumes a designation which implies that he is a legally qualified medical practitioner, breaks the law, and may therefore be visited by appropriate penalties. The term "person," however, takes on a technical meaning when the construction of an Act of Parliament engages the attention of the judicature, and thus, legally, a joint stock company, which to the plain man is merely a number of persons, is not, in the eye of the law, itself a "person." Its legal position is that of a "corporate body," and prohibitions which apply to individuals do not affect it. A company, proverbially, has neither soul that may be saved nor anatomy that may be kicked. In addition, as has now become evident, it may profess itself to be something which no one of its constituents either is, or can legally claim to be. For A, not having fulfilled the legal conditions, to adopt or use such a title as physician or surgeon means the risk of a visit to a police magistrate. But if wise in his generation, A first seeks out six other spirits like unto himself, and then, having formed a joint stock company, announces the combination as "A, B and C Co., Limited, Physicians and Surgeons," there is no one to say him nay, or to question in any degree the strictly legal character of his proceedings.

What is true, as stated above, of the profession and practice of medicine by joint stock companies, is true also of dental practice and of the business of a chemist and druggist. "Medical practitioner," "dentist," "chemist and druggist," all are titles obtained under conditions prescribed by the legislature, and their assumption by unqualified persons carries legal penalties. Yet any one, or all of them, may be adopted by a joint stock company, though not one single member of the company could take this step in his individual capacity. The joint stock company is not a "person," and the legal restraints against "persons" do not apply to it. In this respect it is probable that medical practice and dental practice are even in a worse position than the business of pharmacy. A joint stock company carrying on the business of a chemist and druggist is compelled to employ in each of its shops a person qualified under the Pharmacy Acts, because it has been decided, after a stubborn legal contest, that the "seller" of a poison, is not the person who owns the shop in which the sale takes place, but the individual who hands the goods across the counter. Hence the sale of any statutory poison, or the profession of a legal qualification to carry on the business of a chemist and druggist, does demand the existence and action of an individual qualified under the Pharmacy Acts, even when such business is nominally conducted by a joint stock company. But it may be doubted whether any parallel necessity exists in connection with the practice of medicine. There is no law in this country to prevent anyone from practising medicine and surgery; all that is demanded is that no person shall falsely assume titles which imply the possession of a legal qualification. Hence, apparently, there is nothing to prevent "X, Y, Z Co., Limited, Physicians and Surgeons," from conducting a medical and surgical practice by their own unlicensed hands or by the hands of any other unqualified persons. And, to carry the absurdity a stage further, it is even possible, under the existing law, for a medical practitioner whose name has been erased from the medical register to form a company for the express purpose of securing a style and title the same, nominally, as those of which he has been officially deprived.

Commercial enterprise has already taken advantage of the Companies Act to invade both the art of pharmacy and the practice of dentistry, and it is said that similar methods are in progress in reference to the practice of medicine. In these circumstances it is high time for the legislature to interfere on behalf of honest citizens, and for the protection, more especially, of the poorer members of the community. We are glad, therefore, to note that, on the initiative of Lord Hylton, a Bill has been introduced into the House of Lords for the purpose of making it unlawful for a company formed under the Companies Act to practice as, or carry on the business or profession of, a physician, surgeon, or medical practitioner; and providing a substantial penalty for any "company, director, manager, or other officer" who contravenes this enactment. In short, the object of the Act is to prevent any person doing under the cover of a company what it is at present illegal for him to do as an individual. A similar Act proposes to deal in the same fashion with dental companies, and the Pharmaceutical Society hopes to persuade the legislature to adopt the same attitude toward "company chemists." All these proposals are in the public interest, and merit the hearty support of the medical profession.

